# The Inter-Group Comparison – Intra-Group Cooperation Hypothesis: Comparisons between Groups Increase Efficiency in Public Goods Provision

**DOI:** 10.1371/journal.pone.0056152

**Published:** 2013-02-06

**Authors:** Robert Böhm, Bettina Rockenbach

**Affiliations:** 1 Center for Empirical Research in Economics and Behavioral Sciences, University of Erfurt, Erfurt, Germany; 2 Department of Economics, University of Cologne, Cologne, Germany; University of Leicester, United Kingdom

## Abstract

Identifying methods to increase cooperation and efficiency in public goods provision is of vital interest for human societies. The methods that have been proposed often incur costs that (more than) destroy the efficiency gains through increased cooperation. It has for example been shown that inter-group conflict increases intra-group cooperation, however at the cost of collective efficiency. We propose a new method that makes use of the positive effects associated with inter-group competition but avoids the detrimental (cost) effects of a structural conflict. We show that the mere comparison to another structurally independent group increases both the level of intra-group cooperation and overall efficiency. The advantage of this new method is that it directly transfers the benefits from increased cooperation into increased efficiency. In repeated public goods provision we experimentally manipulated the participants’ level of contribution feedback (intra-group only vs. both intra- and inter-group) as well as the provision environment (smaller groups with higher individual benefits from cooperation vs. larger groups with lower individual benefits from cooperation). Irrespective of the provision environment groups with an inter-group comparison opportunity exhibited a significantly stronger cooperation than groups without this opportunity. Participants conditionally cooperated within their group and additionally acted to advance their group to not fall behind the other group. The individual efforts to advance the own group cushion the downward trend in the above average contributors and thus render contributions on a higher level. We discuss areas of practical application.

## Introduction

It is a fundamental question of society how voluntary cooperation in public goods provision can be promoted to counteract society’s breakdown as envisioned in Hardin’s “tragedy of the commons” [1]. Despite being socially desirable (e.g. reduction of air pollution), provision of public goods (i.e. buying a more environment friendly car) is individually costly and the benefits (i.e. less polluted air) are also shared with non-providers. Therefore, the individual incentives to free-ride on others’ contributions lead to a collectively suboptimal outcome. By now a rich literature in biological and social sciences has identified circumstances fostering cooperation in public goods [2]–[4]. Although the results are promising they are far from satisfactory. In experiments on repeated public goods provision, cooperation typically starts at intermediate levels and declines over time until almost complete free-riding is reached. The most prominent explanation for the observed behavior is based on intra-group comparison in the form of conditional cooperation. Conditional cooperation is the propensity to cooperate as long as others are known (or at least believed) to do the same [5]–[7]. Although the majority of subjects behave in this manner [6], [8], selfishly-biased conditional cooperation and the adaption to some purely selfish actors may explain the decline in overall cooperation in repeated interactions [5], [9].

Since cooperative groups are – *ceteris paribus* – more successful (e.g. grow faster and endure longer) than less cooperative groups, inter-group conflict may have played an important role in the evolution of human cooperation [10], [11]. It has been shown that cooperation within the own group [12], [13] and (costly) norm enforcement (e.g. punishment of non-cooperative group members) [14], [15] increases if the intra-group conflict is embedded in a structural inter-group conflict; the so-called inter-group conflict – intra-group cooperation/cohesion effect. In a similar vein, self-interested actions by group leaders have been shown to decrease in the presence of a rivaling other group [16]. However, in many cases inter-group conflict is a zero-sum game or may even destroy resources because engaging in inter-group conflict generates costs for both the victorious and the inferior group (for instance casualties, monetary resources, damage, and harm in war). Thus, engaging in inter-group conflict by cooperating with the own group members is often inefficient from the collective perspective [17], [18].

It is an open question how one can make use of the positive effect of inter-group processes on the propensity to cooperate efficiently. In the following, we argue that even in the absence of negative inter-group interdependences (i.e., structural inter-group conflicts) inter-group comparison may increase intra-group cooperation, without sacrificing collective efficiency. Our findings extend previous research on public goods provisions by integrating and testing predictions from intra-group as well as inter-group social comparison processes. By doing so, we propose a new mechanism how to increase human cooperation in public goods provision by the means of mere inter-group comparison.

### The Inter-Group Comparison – Intra-Group Cooperation Hypothesis

Adding inter-group comparison to the public good provision problem should not affect players following money-maximizing rationality: They free-ride no matter whether or not another group is present. In the same way, “rational cooperation“[19] should not be affected by the presence of another group: Players cooperate in the repeated game because they believe that others in their own group are altruistic or play a tit-for-tat strategy until an end game effect kicks in. Inequity-averse players [20], [21] decrease cooperation in the case of disadvantageous intra-group comparison (i.e., they contributed more than other group members), and (to a lesser degree) increase their cooperation in the case of advantageous intra-group comparison (i.e. they contributed less than other group members). Hence, players are prone to assimilate to other group members’ contributions [22], [23]. If subjects not only compare their payoff to the members of the own group but also to the members of another group, this may influence their intra-group behavior, even if the other group is structurally independent. It is, however, still an open question whether the payoff comparisons to the members of the other group are weighted differently from the payoff comparisons to the own group members.

Research in social psychology has shown that social comparisons may operate on the inter-individual and intra-group level, but also on the inter-group level [24]. Inter-group comparison processes are of utmost importance to social identity theory [25], [26] and self-categorization theory [27]; see [28] for an overview. The social identity perspective of group formation and intergroup relations proposes that group members are prone to increase positive distinctiveness, that is, they positively maximize relative differences between the outcomes of the own and other groups. Thus, inter-group comparisons may activate a comparative focus [29] that motivates group members to increase the relative (joint) outcome of their own group or to decrease the relative disadvantage in comparison with another group. Hence, even in the absence of a material inter-group conflict that may force *realistic competition* for scarce resources [30], [31], group members may engage in *social competition* to boost their social identity [32] or reduce uncertainty [33]. Following this perspective, subjects should increase cooperation in the case of a disadvantageous inter-group comparison (i.e. the own group’s outcome is lower than the comparison group’s outcome).

What happens if the effects of intra-group and inter-group comparison “pull” in opposite directions? For instance, conditional cooperation may request a high contributor to reduce her contribution, whereas at the same time the low provision level of the own group in comparison to the other group may ask her to increase the contribution. We show that the interplay of these opposed forces results in a mitigation of the overall decline of cooperation. The combination of insights from research in economics and social psychology allows us to hypothesize and show that if groups are not negatively interdependent, increased intra-group cooperation based on mere inter-group comparison may not only be in the interest of each group, but may also be in the collective interest. Therefore, social inter-group interactions by means of mere inter-group comparisons are a powerful method to increase efficiency in human cooperation.

### The Present Research

We conducted a laboratory experiment to investigate the effect of inter-group comparison on cooperation in a repeated linear public goods game. After each round, participants received feedback either about the average contributions of the own group members only (intra-group comparison only; *INTRA* treatment), or about the average contributions of the own *and* another group’s members (intra- and inter-group comparison; *INTER* treatment). Following the proposed inter-group comparison – intra-group cooperation hypothesis, we expected larger intra-group cooperation (i.e., contribution to a public good) if actors are able to compare their own group with another group’s level of cooperation (INTER) than when they may compare their personal with the own group’s level of cooperation only (INTRA). Our design allows for testing the differential effects of advantageous/disadvantageous intra-group and inter-group comparisons on contributions.

Furthermore, we manipulated the provision environment. In one public goods environment (COOP+), provision took place in a smaller group (*n* = 3) with a higher individual return from cooperation (0.7). In the other environment (COOP–) the group was bigger (*n* = 4) and the individual return from cooperation was lower (0.4). In line with previous research [34], [35], we expected larger intra-group cooperation in COOP+ than in COOP–. Moreover, the variation of the provision environment allows for testing the robustness and generality of the proposed inter-group comparison – intra-group cooperation effect under different structural incentives for cooperation.

## Methods

### Ethics Statement

The experiment was conducted at a German University, where institutional review boards or committees are not mandatory (see guidelines of the German Psychological Society: http://www.bdp-verband.org/bdp/verband/ethic.shtml; particularly section C.II.4).

### Participants and Design

Participants were 216 students (68 male, 148 female; *M*_age_ = 22.24, *SD* = 3.24) from various disciplines at the University of Erfurt, Germany. Treatment of participants was in agreement with the ethical guidelines of the German Research Foundation (Deutsche Forschungsgemeinschaft) and the German Psychological Society (DGP). All participants gave their written informed consent to participate voluntarily, assuring them that analyses and publication of experimental data would be without an association to their real identities. Decisions were incentivized; on average, participants earned 7.20 €. Moreover, random assignment to visually separated cubicles and private payment at the end of the experiment preserved the anonymity of participants. The experiment involved no deception of participants. As in other socio-economic experiments, there were no additional ethical concerns.

The experiment used a 2 (level of comparison: INTRA vs. INTER) × 2 (environment: COOP+ vs. COOP–) between-subjects design. There were nine experimental sessions, each consisting of 24 participants. Three sessions (72 participants) were randomly assigned to the INTER / COOP– condition, and two sessions (48 participants) were assigned to each of the other conditions (INTRA / COOP+, INTER / COOP+, and INTRA / COOP–). Due to the intra- and inter-group feedback provided in the INTER treatment, participants of the same intra-group as well as participants of matched groups were interdependent (*n* = 6 in COOP+ and *n* = 8 in COOP–), whereas in the INTRA treatments only participants of the same intra-group were interdependent (*n* = 3 in COOP+ and *n* = 4 in COOP-). Thus, the number of independent observations was 16 in INTRA / COOP+, 8 in INTER / COOP+, 12 in INTRA / COOP–, and 9 in INTER / COOP–.

### Procedure

Participants were recruited to take part in a decision making experiment at the Erfurt laboratory for experimental economics (eLab). The experiment was computerized using the software z-Tree [36]. On arrival, participants drew an index card to determine their cubicle number. Participants received printed instructions, including some examples. The instructions were read aloud by the experimenter. To make sure that all participants understood the structure of the game, they had to correctly answer some control questions before the actual experiment started (see instructions and control questions in the Text S1). Each participant was randomly assigned to a group of three (COOP+) or four (COOP–) members (labeled the blue or the green group) that was matched with a group of the other color. Group assignment and matching remained constant over the entire experiment (*partner matching* protocol). The game consisted of 20 rounds. At the beginning of each round, participants received an endowment of 12 tokens and had to decide individually and independently how many (if any) of these tokens to contribute to a group project. For each token contributed to the group project, each member of the own group (including the contributor) received 0.7 (COOP+) or 0.4 (COOP–) experimental currency units (ECU; thus, it was multiplied by 2.1 or 1.6 and equally distributed among all three or four group members, respectively). For each token kept privately, the individual player only received 1 ECU. After each round, participants were informed about their individual earnings in the respective round and the average contributions of their own group (INTRA and INTER), as well as about the average contributions of the other group (INTER only). The experiment ended with a short post-experimental questionnaire, assessing participants’ demographics. Finally, the participants were informed about their overall payoff and paid privately (exchange rate: 10 ECU = 0.25 €). The whole experiment took about 45 minutes.

### Payoff Function

The individual payoff function is: 
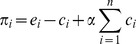
 for player *i* in a group of *n* players, each endowed with *e* tokens, where *c* denotes the number of tokens contributed to the public good and α refers to the amount that each group member (including the contributor) receives for each contributed token.

### Data Analysis

For non-parametric tests, we report two-tailed *p*-values of exact tests in all analyses to account for the relatively small number of independent observations. Additionally, we provide *r*-values as effect size approximations. Parametric feedback analyzes were computed using the statistical package *nlme* [37] in the R environment [38]. For analyzes of feedback in the INTRA treatment, intra-groups (*N* = 28) and participants (*N* = 96) were treated as random effects to control for their interdependent error terms (*random intercept model*; [39]). Similarly, for feedback analyzes in the INTER treatment, matched groups (*N* = 17), intra-groups (*N* = 34), and participants (*N = *120) were treated as random effects.

## Results

**Figure 1:**
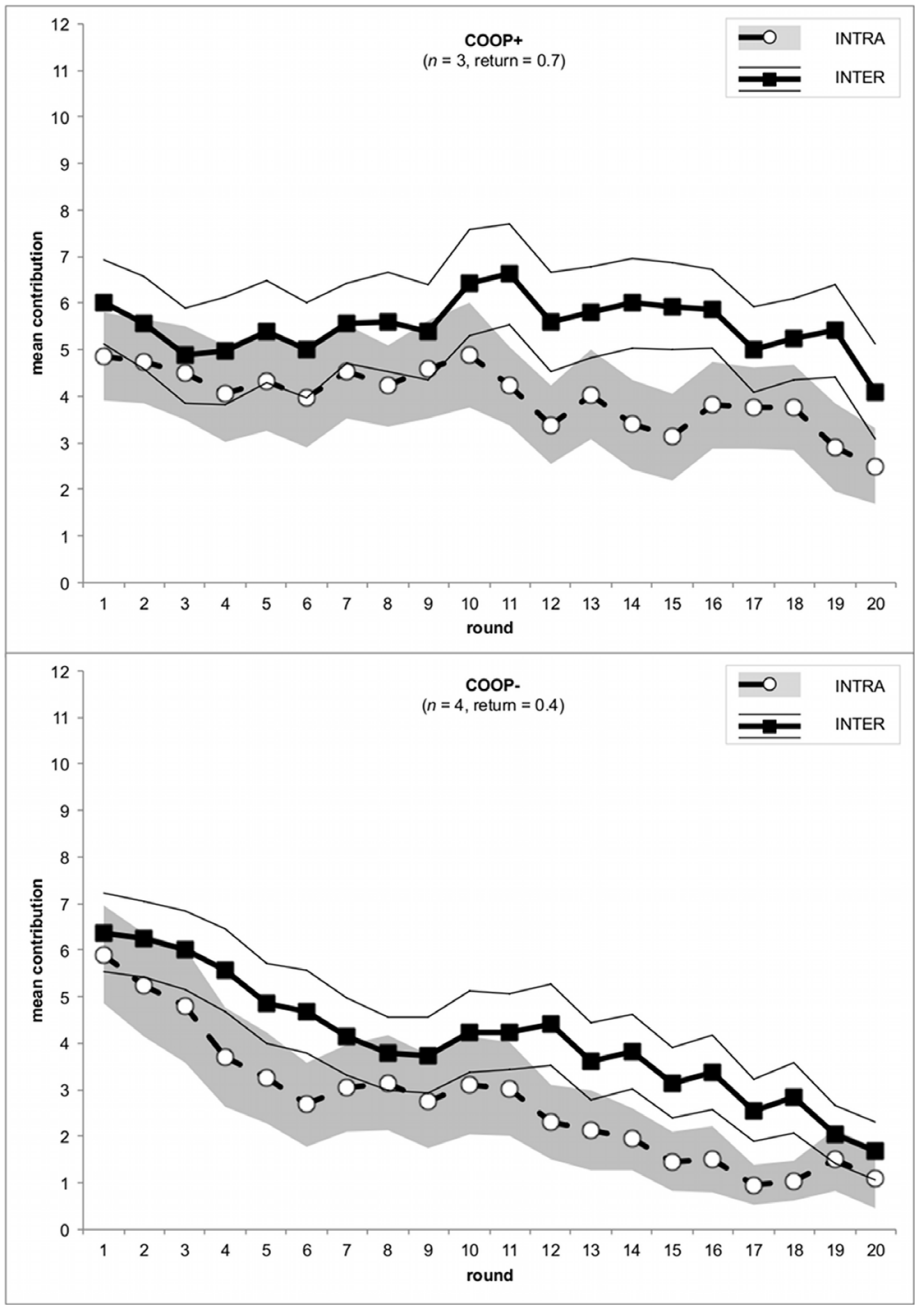
Mean contributions per round by comparison and environment treatments. Areas around mean values indicate 95% confidence intervals.

Our data provide clear evidence for the inter-group comparison – intra-group cooperation hypothesis (see Dataset S1). Overall, contributions in INTER were about 40% higher than in INTRA (Mann-Whitney U test: *z* = -2.81, *p* = .004, *r* = -.42). This result holds in each of the provision environments COOP+ and COOP– (Mann-Whitney U test: COOP+: *z* = -2.51, *p* = .010, *r* = -.51; COOP–: *z* = -2.49, *p* = .012, *r* = -.54). Figure 1 visualizes this result by depicting the mean individual contributions and its 95% confidence intervals per round and comparison treatment, separately for each provision environment treatment. Table 1 displays the mean values and standard deviations of all conditions at the appropriate level of comparison. As further expected, overall contributions in COOP+ were higher than in COOP– (Mann-Whitney U test: *z* = -2.66, *p* = .007, *r* = -.40).

**Table 1:** Mean values and standard deviations (in brackets) of the first round, the first 10 rounds, the last 10 rounds, and overall contributions by condition.

ConditionContribution	INTRA		INTER	
	COOP+	COOP–	COOP+	COOP–
First round	4.85 (3.35)	5.90 (3.71)	6.02 (3.21)	6.37 (3.61)
Round 1–10	4.47 (1.71)	3.77 (2.20)	5.49 (1.30)	4.97 (1.40)
Round 11–20	3.49 (1.65)	1.70 (1.23)	5.56 (1.35)	3.17 (1.42)
Overall	3.98 (1.58)	2.73 (1.69)	5.52 (1.17)	4.07 (1.31)

INTRA: intra-group comparison only. INTER: intra- and inter-group comparison. COOP+: public good with *n* = 3 and individual return from cooperation = 0.7. COOP–: public good with *n* = 3 and individual return from cooperation = 0.4. Reported are mean values on the level of independent observations. Thus, values of first round contributions are mean values on the individual level. Values of contributions in round 1–10, round 11–20, and overall are mean values on the level of intra-groups (INTRA) or on the level of matched groups (INTER).

### Development of Contributions

As Table 1 shows, in the first round participants contributed about half of their endowment irrespective of the comparison treatment, *F*(1, 215) = 2.91, *p* = .089, and the environment treatment, *F*(1, 215) = 2.09, *p* = .149. Overall, we observe the usual decline in contributions. Contributions in the last 10 rounds were significantly lower than in the first 10 rounds, Wilcoxon signed-rank test: *z* = -4.71, *p*<.001, *r* = -.70. However, when evaluating the experimental conditions separately this result only holds in three of the four conditions. In INTER / COOP+ where participants could compare their own group’s average contributions to another group in an environment with high cooperative incentives, contributions exhibited no significant decline over the rounds, but remained rather constant, Wilcoxon signed-rank test: *z* = -.14, *p* = .945, *r* = -.05.

### Reactions to Intra- and Inter-Group Feedback

To study the subjects’ reactions to feedback, we investigated the contribution change (contribution in the actual round minus contribution in the previous round) in different mixed-effects models to account for observations’ interdependence, while controlling for the provision environment (see Table 2). As predictors we used the deviation of the subject’s contribution from the average of other members of the own group (own contribution minus average contributions of other group members) and additionally in INTER the contribution deviation of the own group’s average from the other group’s average (own group average contribution minus other group average contributions).

**Table 2:** Parameter estimates of mixed-effects models predicting contribution change.

Predictor		Model					
		1				2	
		INTRA		INTER		INTER	
		Estimate	t-value	Estimate	t-value	Estimate	t-value
(Intercept)		−0.256	−1.99	−0.247	−1.85	−.056	−0.31
		(0.129)		(0.133)		(0.184)	
Environment		0.139	0.77	0.143	0.68	0.162	0.75
		(0.182)		(0.211)		(0.215)	
Intra-group comparison	Overall	−0.564	−28.59***	−0.549	−30.96***	−	−
		(0.020)		(0.018)			
	Positive (disadvantageous)	−	−	−	−	−0.666	−21.13***
						(0.032)	
	Negative (advantageous)	−	−	−	−	−0.421	−12.05***
						(0.035)	
Inter-group comparison	Overall	−	−	−0.219	−9.60***	−	−
				(0.023)			
	Positive (advantageous)	−	−	−	−	−0.149	−3.54**
						(0.042)	
	Negative (disadvantageous)	−	−	−	−	−0.276	−6.50***
						(0.042)	
Observations [subjects/intra-groups/matched groups]		1824 [96/28/−]		2280 [120/34/17]		2280 [120/34/17]	
REML model fit: AIC/BIC		9020/9053		11446/11492		11433/11491	

In model 1 INTRA subjects and intra-groups were treated as random effects, whereas in all other models subjects, intra-groups, and matched groups were treated as random effects. The presented models are superior regarding AIC/BIC to other model specifications (e.g. including interaction terms). REML = restricted maximum likelihood. AIC = Akaike information criterion. BIC = Bayesian information criterion. * *p*<.01, ** *p*<.001, *** *p*<.0001.

When only intra-group comparison was available (INTRA), the deviation from the own group’s average significantly predicted the change in cooperation, *b* = -.56, *SE* = .02, *t*_1727_ = -28.59, *p*<.0001 (model 1 INTRA in Table 2). When the deviation was positive, i.e. the subject contributed more than the average of the other members of the own group, the negative estimate predicts a reduction in contribution, while the subjects increased the contribution when having contributed less than the other group members. This indicates conditional cooperation. When both intra- and inter-group comparison was available (INTER), both the intra- and the inter-group deviation significantly predicted changes of contributions, *b* = -.55, *SE* = .02, *t*_2158_ = -30.96, *p*<.0001 and *b* = -.22, *SE* = .02, *t*_2158_ = -9.60, *p*<.0001, respectively (model 1 INTER in Table 2). This shows that subjects not only conditionally cooperate within their own group, but also want their group to be ahead of the other group, yielding support for the inter-group comparison – intra-group cooperation hypothesis.

**Figure 2:**
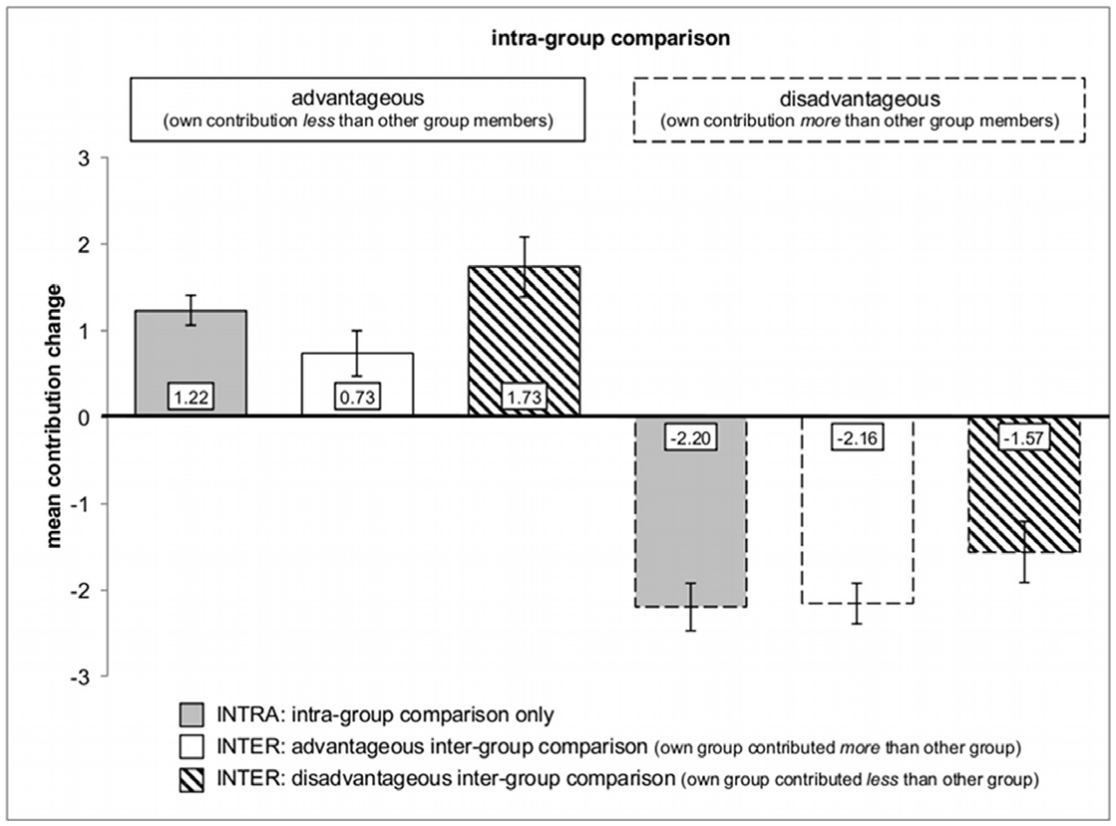
Mean contribution change by advantageous and disadvantageous intra- and inter-group comparison. Error bars indicate 95% confidence intervals.

But how do subjects behave if these two motives – conditional cooperation within the own group and being ahead of the other group – are in conflict? How does a subject react if conditional cooperation calls to reduce cooperation, but at the same time the desire to be ahead of the other group calls for increasing cooperation? To answer these questions we distinguish between positive and negative deviations in both the intra- and the inter-group comparison (model 2 in Table 2). In the case of intra-group comparison a positive deviation indicates a disadvantage (i.e. “I have contributed *more* than other group members.”), while in the case of inter-group comparison it indicates an advantage (i.e. “My group has contributed *more* than the other group.”). In contrast, a negative deviation in the case of intra-group comparison indicates an advantage (i.e. “I have contributed *less* than other group members.”), while in the case of inter-group comparison it indicates a disadvantage (i.e. “My group has contributed *less* than the other group.”). In the spirit of inequity aversion [21] the decrease in reaction to a disadvantageous intra-group comparison is stronger than the increase in reaction to an advantageous comparison, *b* = -.67, *SE* = .03, *t*_2156_ = -21.13, *p*<.0001 and *b* = -.42, *SE* = .04, *t*_2156_ = -12.05, *p*<.0001, respectively (see Figure 2: white and hatched bars on the left-hand side have smaller absolute values than on the right-hand side). Adding inter-group comparison has two remarkable effects. First, the increase in contribution triggered by a comparison group with higher contributions is much stronger than the decrease in contribution when being ahead of the other group, *b* = -.28, *SE* = .04, *t*_2156_ = -6.50, *p*<.0001 and *b* = -.15, *SE* = .04, *t*_2156_ = -3.54, *p* = .0004, respectively (see Figure 2: hatched bars have greater values than white bars). And second, the contribution adaptation due to the intra-group comparison is always stronger than the one based on the inter-group comparison, resulting in a net effect in the direction of conditional cooperation (see Figure 2: the adaption according to advantageous intra-group comparison is always positive and the adaption according to disadvantageous intra-group comparison is always negative, irrespective of the inter-group comparison). Hence, the inter-group comparison may accelerate conditional cooperation among the low contributors of the group, but most importantly it dampens the downward trend in the high contributions. The desire to be ahead of the other group cushions the downward trend in the contributions of subjects contributing above average (see Figure 2: grey bar on the right-hand side has smaller value than hatched bar).

## Discussion

The present research presents a new mechanism to increase cooperation and efficiency in public goods provision by integrating research on intra-group [20], [21] and inter-group social comparisons [25]–[29]. The mere inter-group comparison suffices to increase intra-group cooperation and overall efficiency. We found higher contributions to a public good in the presence of both intra-group and inter-group feedback of average group contributions than in the presence of intra-group feedback only. The effect appeared across two provision environments with differing individual incentives for intra-group cooperation, supporting its robustness and generality. Furthermore, analyzes provided evidence that the overall level of cooperation may be explained by the combination of intra-group conditional cooperation and a desire to be ahead of the other group.

The results also contribute to the empirical literature on the inter-group conflict – intra-group cooperation effect [12]–[16], that is, an increase of intra-group cooperation in the presence of a structural inter-group conflict. In contrast to this well-supported phenomenon, however, mere inter-group comparison does not increase cooperation at the cost of collective efficiency. Rather, it is in the interest of all individuals, irrespective of group membership, to maximize contributions to the (intra-group) public good. Therefore, the inter-group comparison – intra-group cooperation hypothesis proposes an efficiency-enhancing alternative to increase long-term human cooperation.

### Practical Implications

Social comparisons are made frequently in everyday life [40]. It has been shown that inter-individual and intra-group social comparisons may increase individual performance, for instance in task performance [41], [42], academic performance [43], and sports performance [44], but also different kinds of prosocial behavior [45]–[48]. Our results suggest that social comparisons not only on the individual level but also on the group-level might further increase such effects. For instance, providing feedback about large-scale prosocial behavior (e.g., charity donations) of other groups (organizations, countries, etc.) might help to increase individual cooperation/prosocial behavior in the own group in order to receive a positive inter-group comparison. In a similar vein, if the level and success of environmental protection of different villages or towns may be quantified and made publically available (e.g. CO2 emissions), this might motivate further activities on the individual and group level in order to improve one’s own “group’s” ranking. Also, because the success of a company requires coordination and cooperation among employees of its sub-units, the company could provide feedback not only on the individual level [46], [47] but also on the sub-units’ level of cooperation. This might increase (intra-sub-unit) organizational collaboration without any additional costs for monetary incentives (in order to induce a resource conflict among the sub-units).

## Supporting Information

Text S1 **Instructions and control questions.**
(PDF)

Dataset S1 (CSV)
