# Supplementary material for: The Inter-Group Comparison – Intra-Group Cooperation Hypothesis: Comparisons between Groups Increase Efficiency in Public Goods Provision
Source: PLoS One. 2013 Feb 6;8(2):e56152. doi: 10.1371/journal.pone.0056152 (PMC3566068; doi:10.1371/journal.pone.0056152)
Supplement: Text S1 — Instructions and control questions. (PDF) [file pone.0056152.s001.pdf]

Note: Instructions were exactly the same in INTRA and INTER except for the grey-shaded text, which was only given in the INTER treatment

Instructions

Group Assignment

- All participants will be assigned randomly either to a **blue or green group**, each with 4 members.
- Each blue group is allocated to a green group.
- You will neither know the personal identities of your own group's members, nor of the other group that is allocated to your group.
- The group assignment and allocation remains constant over the experiment. This means that the members of your group and of the other group that is allocated to your group are the same persons during the entire experiment.

Rounds

- The experiment has **20 rounds**, each consisting of the decision making task described below.
- Each participant receives an **endowment of 12 points** in every round.

Decision Making Task

- You may contribute any integer amount from 0 to 12 from your endowment to a group project. Points not contributed to the group project are kept personally.

Group Project

- Every member of your own group may contribute to the project and all **members of the own group** benefit from contributions to the project.
- **More precisely:** Each point allocated to the project by members of the own group will be **multiplied by 1.6** and **distributed equally among all 4 members of the own group**. **Hence, each member of the own group receives 0.4 points for each point contributed by members of the own group.**

Profit for each member of the own group from the group project = **0.4** x sum of contributions by **all members of the own group**

Points Kept

- **You keep every point of your endowment that is not contributed to the group project privately.**
- Only you profit from kept points.

Profit of each Round

- The profit per round for each participant is the sum of points kept and the profit from the group project.

Profit per round = points kept + **0.4** x sum of contributions by **all members of the own group** to group project

Example

Group	**Blue**				**Green**			
Member	**B1**	**B2**	**B3**	**B4**	**G1**	**G2**	**G3**	**G4**
Endowment	**12**	**12**	**12**	**12**	**12**	**12**	**12**	**12**
Contribution to group project	8	4	6	12	0	12	10	5
Sum of contributions	**30**				**27**			
Profit from group project	**0.4 x 30 = 12**				**0.4 x 27 = 10.8**			
Points kept	4	8	6	0	12	0	2	7
Profit of round	16	20	18	12	22.8	10.8	12.8	17.8

Information after each round

- After each round you receive the following information regarding the decisions:
  - average contribution to the group project by members of your group,
  - average contribution to the group project by members of the other group, which is allocated to your group,
  - your profit in the respective round.
- After 20 rounds your receive the information about your aggregated profit.

Payment

- All points earned are converted at the end of the experiment with **10 points = 0.25 €**.
- All participants are called individually and the profit is paid privately and anonymously in cash. Your will not know the profit of other participants and other participants will not know your profit.

Note: Control questions were presented on the computer screen. After participants gave their response, it was shown whether this was correct or wrong. If the response was wrong, they had to repeat it until the correct answer was given.

Control Questions

Q1. What is the individual endowment for each player in each round?

Correct answer: 12

Q2. What is the payoff from points kept for player B2 (see table in instructions)?

Correct answer: 8

Q3. What is the payoff from the group project for player B2 (see table in instructions)?

Correct answer: 12
